# Supplementary figures and images for: Network neighborhood operates as a drug repositioning method for cancer treatment
Source: PeerJ. 2023 Jul 10;11:e15624. doi: 10.7717/peerj.15624 (PMC10340098; doi:10.7717/peerj.15624)

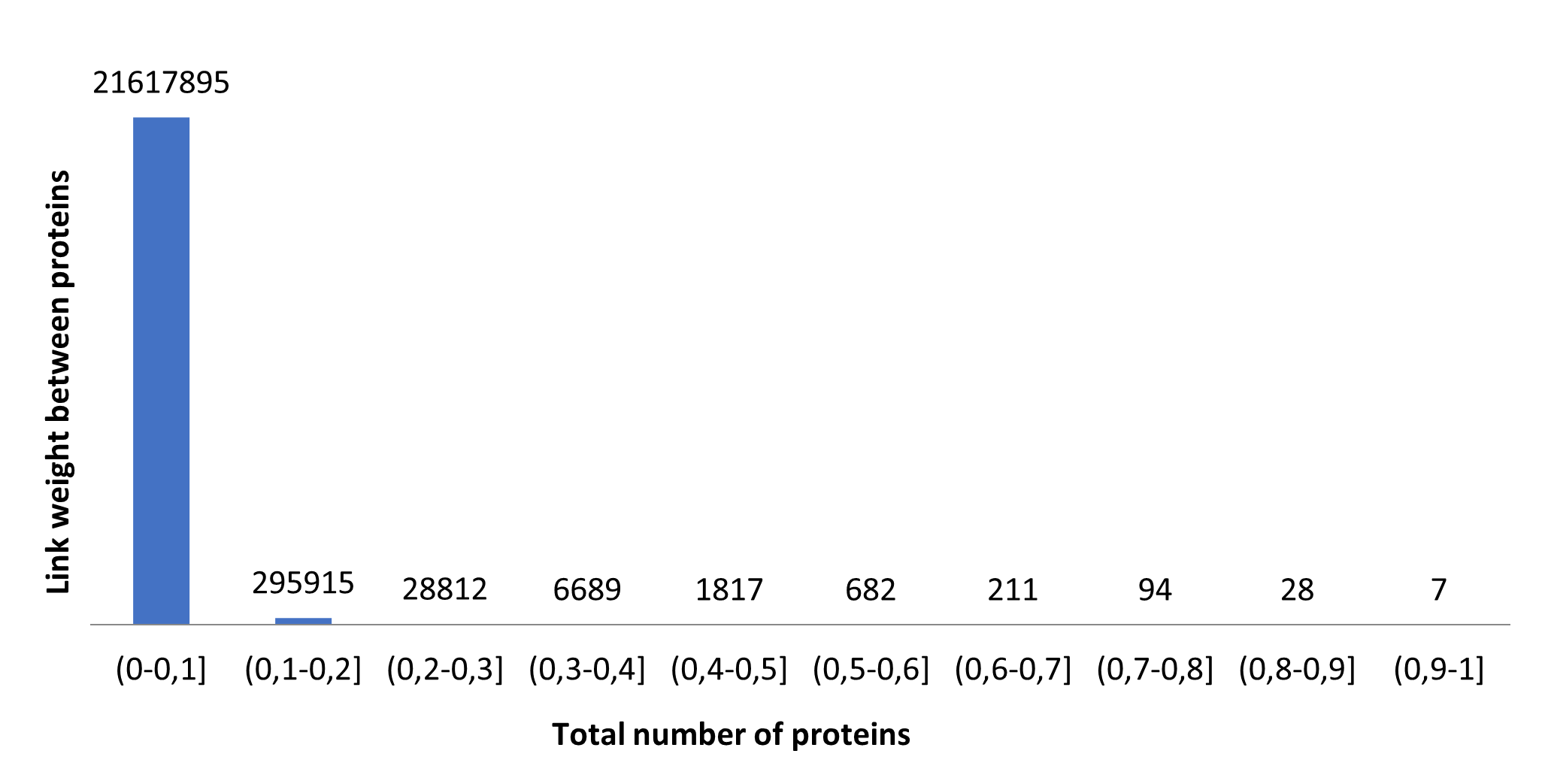

Supplement: Supplemental Information 1 — The x-axis shows the specific weight ranges. The y-axis shows the total number of edges (links) in the corresponding weight range. [file peerj-11-15624-s001.png]
