# Supplementary material for: Network neighborhood operates as a drug repositioning method for cancer treatment
Source: PeerJ. 2023 Jul 10;11:e15624. doi: 10.7717/peerj.15624 (PMC10340098; doi:10.7717/peerj.15624)
Supplement: Supplemental Information 2 — The x-axis shows the possible drug dosages. The y-axis shows AUC value of the corresponding network metric. [file peerj-11-15624-s002.pdf]

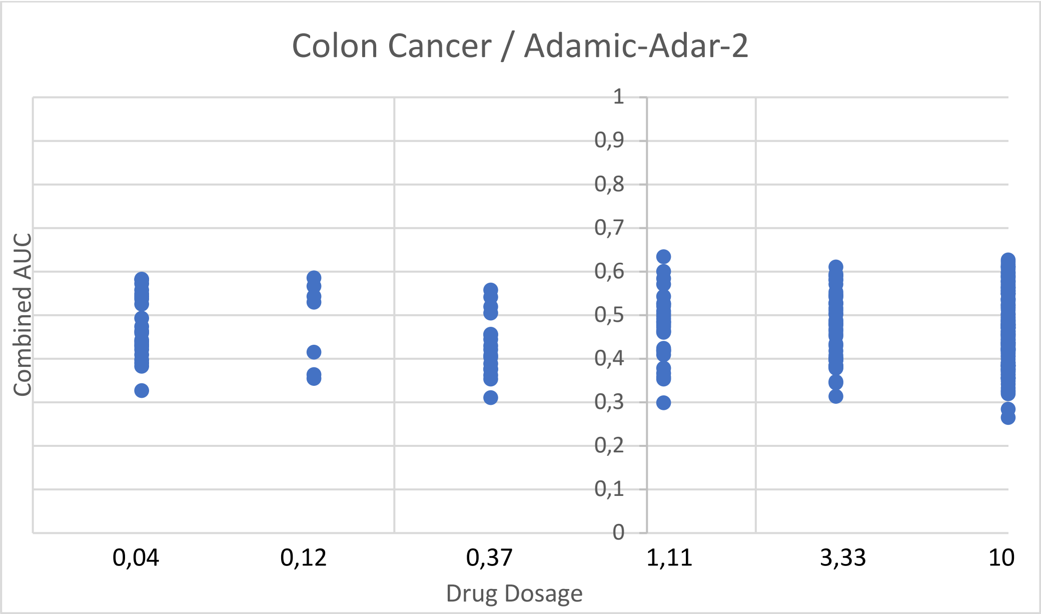

a) Distribution of AUC values with corresponding drug dosages in colon cancer (using Adamic-Adar-2)

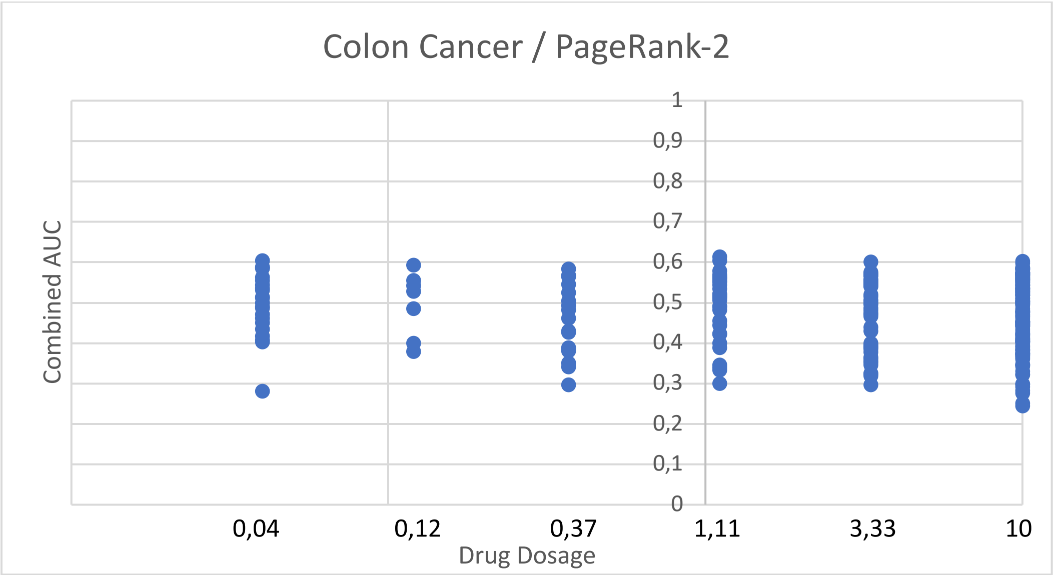

b) Distribution of AUC values with corresponding drug dosages in colon cancer (using PageRank-2)

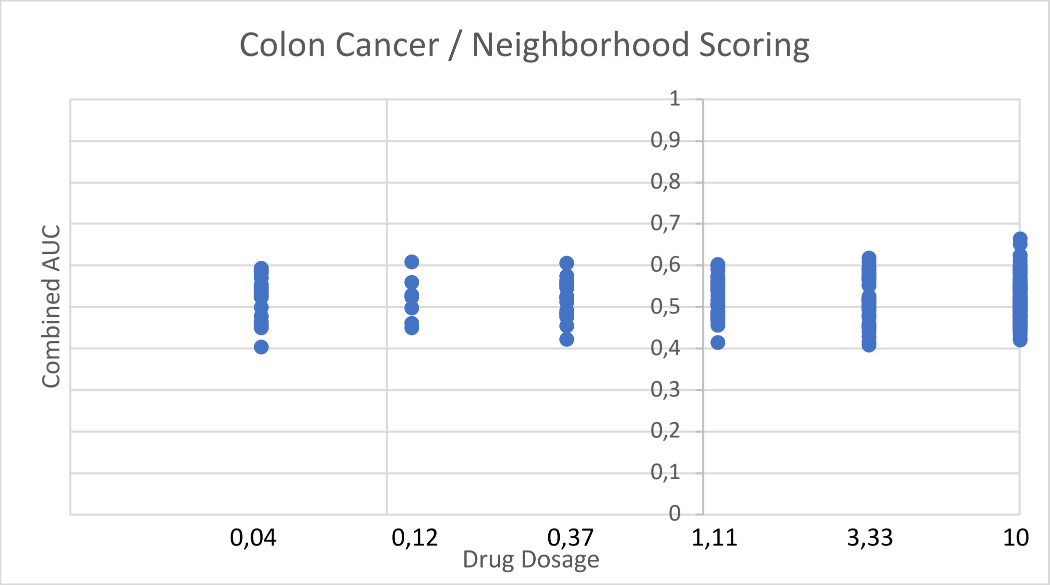

c) Distribution of AUC values with corresponding drug dosages in colon cancer (using Neighborhood Scoring)

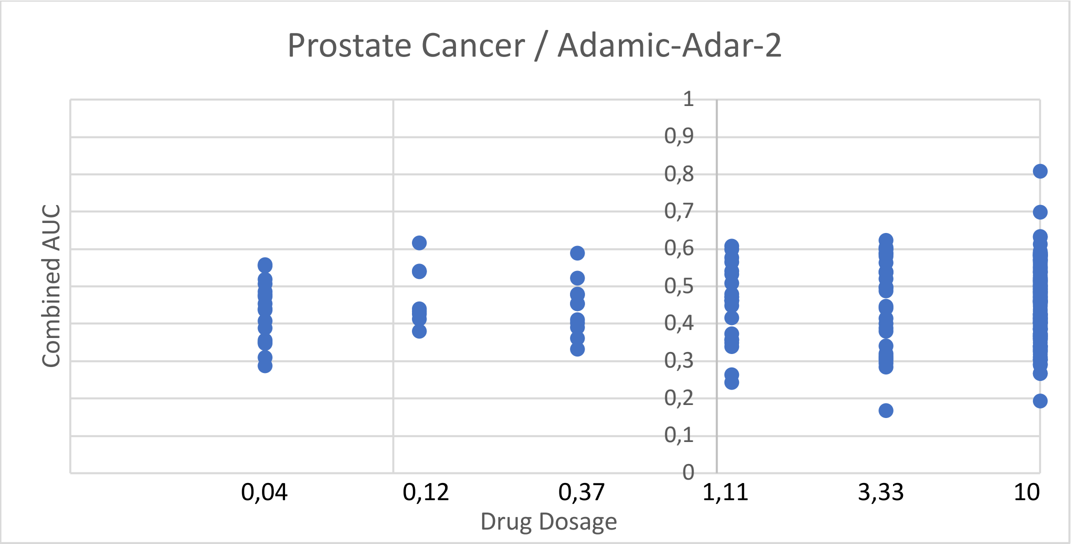

d) Distribution of AUC values with corresponding drug dosages in prostate cancer (using Adamic-Adar-2)

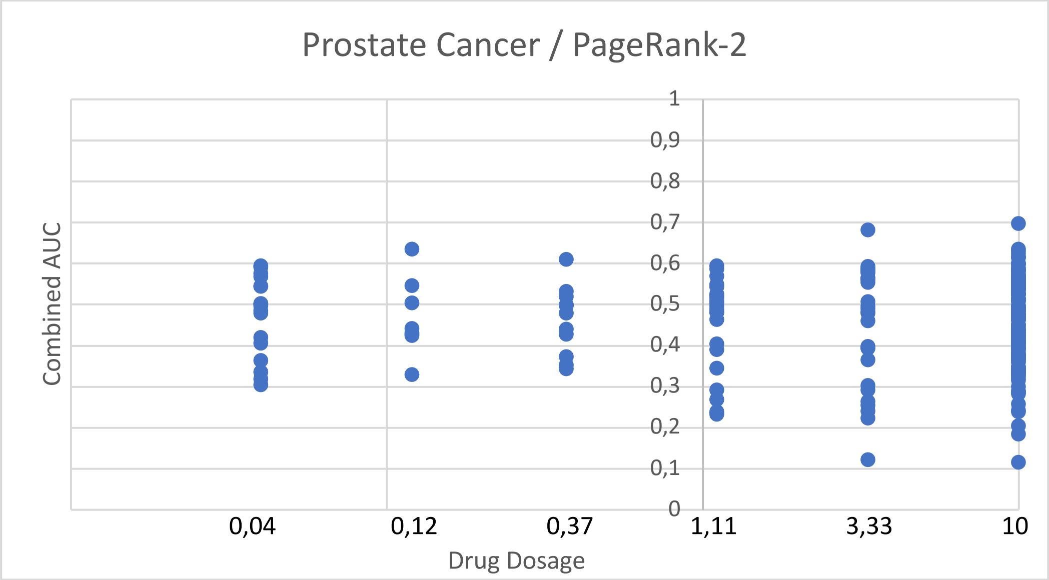

e) Distribution of AUC values with corresponding drug dosages in prostate cancer (using PageRank-2)

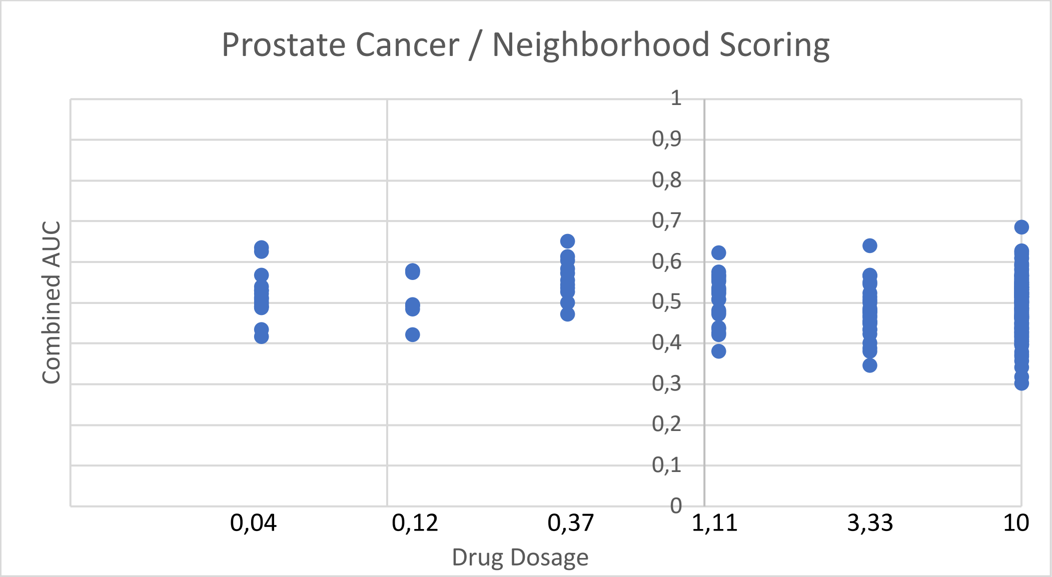

f) Distribution of AUC values with corresponding drug dosages in prostate cancer (using Neighborhood Scoring)

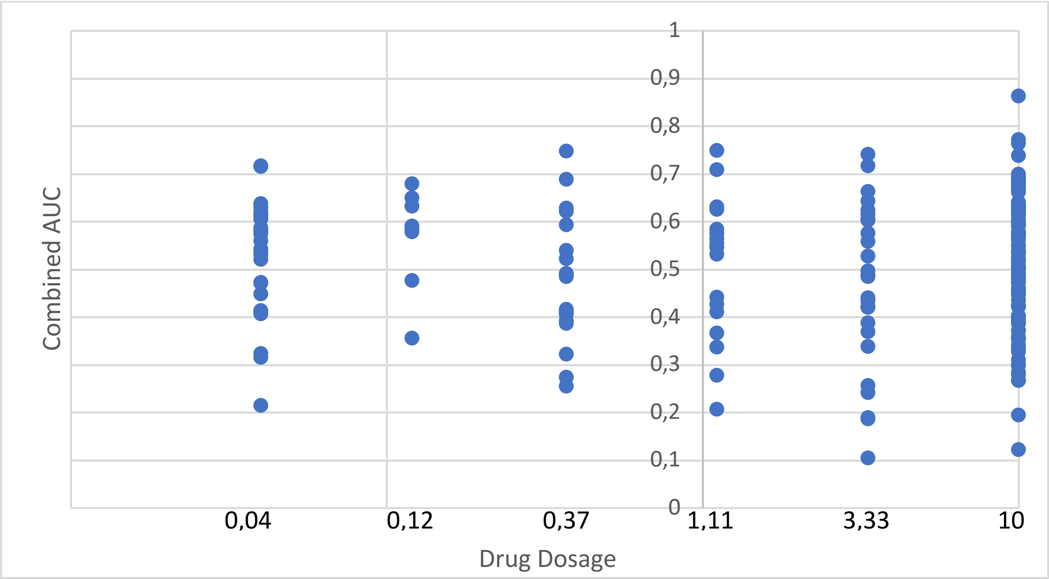

g) Distribution of AUC values with corresponding drug dosages in melanoma (using Adamic-Adar-2)

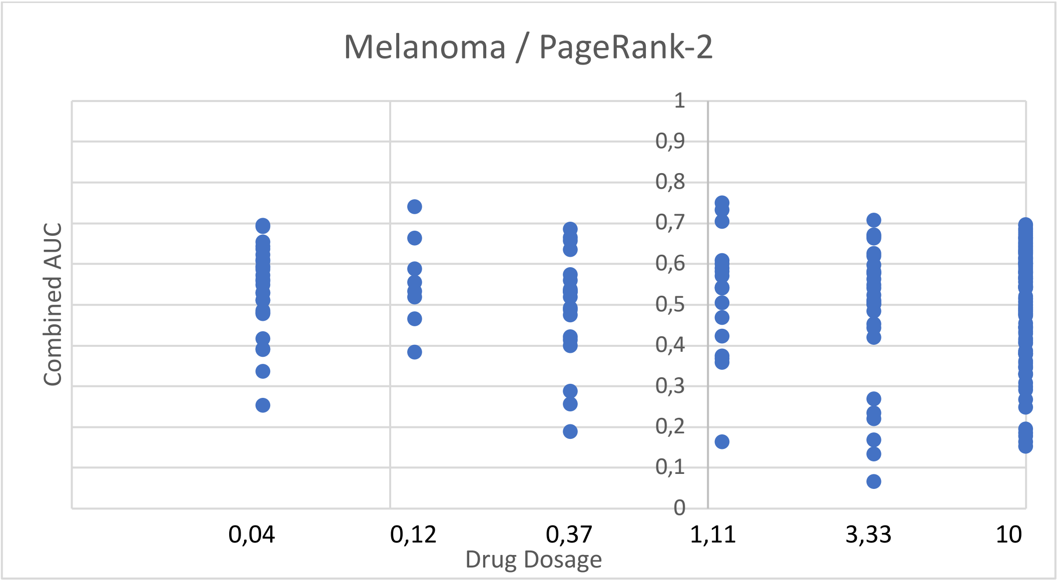

h) Distribution of AUC values with corresponding drug dosages in melanoma (using PageRank-2)

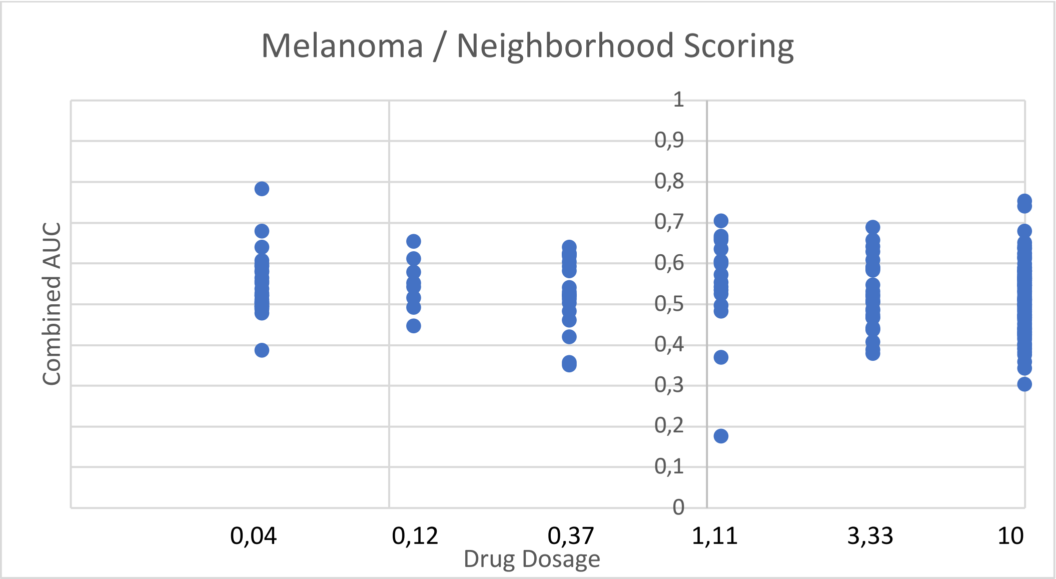

i) Distribution of AUC values with corresponding drug dosages in melanoma (using Neighborhood Scoring-2)
