# Supplementary material for: Network neighborhood operates as a drug repositioning method for cancer treatment
Source: PeerJ. 2023 Jul 10;11:e15624. doi: 10.7717/peerj.15624 (PMC10340098; doi:10.7717/peerj.15624)
Supplement: Supplemental Information 6 — Yellow marked drugs are matched with the top prediction of the current study. [file peerj-11-15624-s006.docx]

**Supplementary Table 4:** SAveRUNNER results for colon cancer. Yellow marked drugs are matched

with the top prediction of the current study.

| **Drug** | **Proximity** | **p-value** | **Similarity** | **Adjusted Similarity** |
| --- | --- | --- | --- | --- |
| temozolomide | 0,0000 | 0,0035 | 1,0000 | 0,9999 |
| acetazolamide | 0,3750 | 0,0001 | 0,8393 | 0,9986 |
| crizotinib | 0,5000 | 0,0080 | 0,7857 | 0,9961 |
| fulvestrant | 0,5000 | 0,0000 | 0,7857 | 0,9961 |
| vemurafenib | 0,5000 | 0,0001 | 0,7857 | 0,9961 |
| bicalutamide | 0,6000 | 0,0039 | 0,7429 | 0,9912 |
| cabozantinib | 0,6000 | 0,0200 | 0,7429 | 0,9912 |
| celecoxib | 0,6000 | 0,0000 | 0,7429 | 0,9912 |
| temsirolimus | 0,6000 | 0,0169 | 0,7429 | 0,9912 |
| osimertinib | 0,6667 | 0,0466 | 0,7143 | 0,9847 |
| palbociclib | 0,6667 | 0,0004 | 0,7143 | 0,9847 |
| tadalafil | 0,6667 | 0,0299 | 0,7143 | 0,9847 |
| sn-38 | 0,6818 | 0,0000 | 0,7078 | 0,9827 |
| ibuprofen | 0,6875 | 0,0001 | 0,7054 | 0,9818 |
| chrysin | 0,6970 | 0,0000 | 0,7013 | 0,9804 |
| erlotinib | 0,7000 | 0,0040 | 0,7000 | 0,9799 |
| gefitinib | 0,7143 | 0,0069 | 0,6939 | 0,9774 |
| naproxen | 0,7143 | 0,0371 | 0,6939 | 0,9774 |
| nilotinib | 0,7143 | 0,0140 | 0,6939 | 0,9774 |
| primaquine | 0,7143 | 0,0244 | 0,6939 | 0,9774 |
| vandetanib | 0,7143 | 0,0038 | 0,6939 | 0,9774 |
| irinotecan | 0,7273 | 0,0015 | 0,6883 | 0,9749 |
| etoposide | 0,7500 | 0,0144 | 0,6786 | 0,9698 |
| troglitazone | 0,7600 | 0,0005 | 0,6743 | 0,9673 |
| imatinib | 0,8000 | 0,0110 | 0,6571 | 0,9549 |
| sorafenib | 0,8095 | 0,0021 | 0,6531 | 0,9514 |
| aspirin | 0,8125 | 0,0281 | 0,6518 | 0,9502 |
| dabrafenib | 0,8333 | 0,0463 | 0,6429 | 0,9414 |
| tamoxifen | 0,8333 | 0,0013 | 0,6429 | 0,9414 |
| thalidomide | 0,8462 | 0,0311 | 0,6374 | 0,9352 |
| enoxolone | 0,8846 | 0,0115 | 0,6209 | 0,9128 |
| ponatinib | 0,8947 | 0,0130 | 0,6165 | 0,9059 |
