# Supplementary material for: Network neighborhood operates as a drug repositioning method for cancer treatment
Source: PeerJ. 2023 Jul 10;11:e15624. doi: 10.7717/peerj.15624 (PMC10340098; doi:10.7717/peerj.15624)
Supplement: Supplemental Information 7 — Yellow marked drugs are matched with the top prediction of the current study. [file peerj-11-15624-s007.docx]

**Supplementary Table 5:** SAveRUNNER results for prostate cancer. Yellow marked drugs are matched

with the top prediction of the current study.

| **Drug** | **Proximity** | **p-value** | **Similarity** | **Adjusted Similarity** |
| --- | --- | --- | --- | --- |
| naftopidil | 0,0000 | 0,0140 | 1,0000 | 0,9999 |
| crizotinib | 0,7500 | 0,0097 | 0,6786 | 0,9701 |
| epirubicin | 0,7500 | 0,0060 | 0,6786 | 0,9701 |
| etoposide | 0,7500 | 0,0001 | 0,6786 | 0,9701 |
| erlotinib | 0,8000 | 0,0000 | 0,6571 | 0,9553 |
| temsirolimus | 0,8000 | 0,0109 | 0,6571 | 0,9553 |
| ciprofloxacin | 0,8333 | 0,0143 | 0,6429 | 0,9417 |
| lapatinib | 0,8333 | 0,0031 | 0,6429 | 0,9417 |
| gefitinib | 0,8571 | 0,0238 | 0,6327 | 0,9298 |
| pioglitazone | 0,8571 | 0,0261 | 0,6327 | 0,9298 |
| sorafenib | 0,8571 | 0,0003 | 0,6327 | 0,9298 |
| imatinib | 0,8667 | 0,0006 | 0,6286 | 0,9245 |
| doxazosin | 0,8750 | 0,0006 | 0,6250 | 0,9194 |
| enzalutamide | 0,8750 | 0,0114 | 0,6250 | 0,9194 |
| flutamide | 0,8750 | 0,0211 | 0,6250 | 0,9194 |
| rosiglitazone | 0,8889 | 0,0050 | 0,6190 | 0,9104 |
| gdc-0941 | 0,9091 | 0,0236 | 0,6104 | 0,8957 |
| sn-38 | 0,9091 | 0,0000 | 0,6104 | 0,8957 |
| ponatinib | 0,9474 | 0,0374 | 0,5940 | 0,8618 |
| cinnarizine | 0,9545 | 0,0002 | 0,5909 | 0,8546 |
| troglitazone | 0,9600 | 0,0000 | 0,5886 | 0,8488 |
| irinotecan | 1,0000 | 0,0008 | 0,5714 | 0,8008 |
| pi-103 | 1,0000 | 0,0429 | 0,5714 | 0,8008 |
| quinine | 1,0000 | 0,0183 | 0,5714 | 0,8008 |
| doxorubicin | 1,0556 | 0,0050 | 0,5476 | 0,7166 |
| tamoxifen | 1,0667 | 0,0036 | 0,5429 | 0,6974 |
| chrysin | 1,1515 | 0,0076 | 0,5065 | 0,5316 |
| finasteride | 1,1667 | 0,0428 | 0,5000 | 0,5000 |
