# Supplementary material for: Network neighborhood operates as a drug repositioning method for cancer treatment
Source: PeerJ. 2023 Jul 10;11:e15624. doi: 10.7717/peerj.15624 (PMC10340098; doi:10.7717/peerj.15624)
Supplement: Supplemental Information 8 — Yellow marked drugs are matched with the top prediction of the current study. [file peerj-11-15624-s008.docx]

**Supplementary Table 6:** SAveRUNNER results for melanoma. Yellow marked drugs are matched

with the top prediction of the current study.

| **Drug** | **Proximity** | **p-value** | **Similarity** | **Adjusted similarity** |
| --- | --- | --- | --- | --- |
| avl-292 | 0,0000 | 0,0131 | 1,0000 | 0,9992 |
